# Trends in clinical characteristics, medication use, and glycemic control in insulin‐treated patients with type 1 and type 2 diabetes in Finland in 2012–2019: Nationwide real‐world evidence study

**DOI:** 10.1111/1753-0407.13491

**Published:** 2024-01-25

**Authors:** Leo Niskanen, Minna Hannula, Kai Kysenius, Saara Kaijala, Mariann I. Lassenius, Timo T. Valle

**Affiliations:** ^1^ Päijät‐Häme Central Hospital, Department of Internal Medicine, Lahti, Finland, Eira Hospital Helsinki Finland and University of Eastern Finland, Institute of Clinical Medicine Kuopio Finland; ^2^ Sanofi Espoo Finland; ^3^ Medaffcon Oy Espoo Finland; ^4^ Boa‐8 Oy Helsinki Finland

## Abstract

**Aims:**

To describe the clinical characteristics and medication purchases of insulin‐treated adults in Finland at index (January 1, 2012 or first insulin purchase) and December 31, 2019. Additionally, to describe basal insulin (BI) treatment patterns and associated changes in hemoglobin A1c (HbA1c) values.

**Materials and Methods:**

In this descriptive study using nationwide registries, we included adults with at least two reimbursed insulin purchases within 12 months of the first purchase between January 1, 2012 and December 31, 2019. We formed four study groups: type 1 diabetes (T1D) and type 2 diabetes (T2D)‐diagnosed people who were further divided into prevalent or naïve users (start of insulin use before or after January 1, 2012). Insulin treatment patterns were estimated from medication purchase data and glycemic control from HbA1c results.

**Results:**

Out of 145 020 people included, 34 359 had T1D and 110 661 T2D. By 2019, in parallel with the adaptation of new noninsulin medications, second‐generation basal insulin (BI) analogues were adopted by 45.9% and 21.1% of prevalent T1D and T2D users. At index, HbA1c target (≤53 mmol/mol) was reached by 17% and 35% of T2D naïve and prevalent users, respectively, and by 17% of T1D prevalent users. At study end, the target was reached respectively by 41%, 34%, and 22% of insulin users. Insulin initiation improved and discontinuation worsened glycemic control in T2D, with lesser effects seen after treatment gaps or switches between BIs.

**Conclusions:**

Our study showed that glycemic control in insulin users has remained stable or improved between 2012 and 2019 despite aging population and in parallel with introduction of new treatment options, providing valuable insight into Finnish national diabetes care.

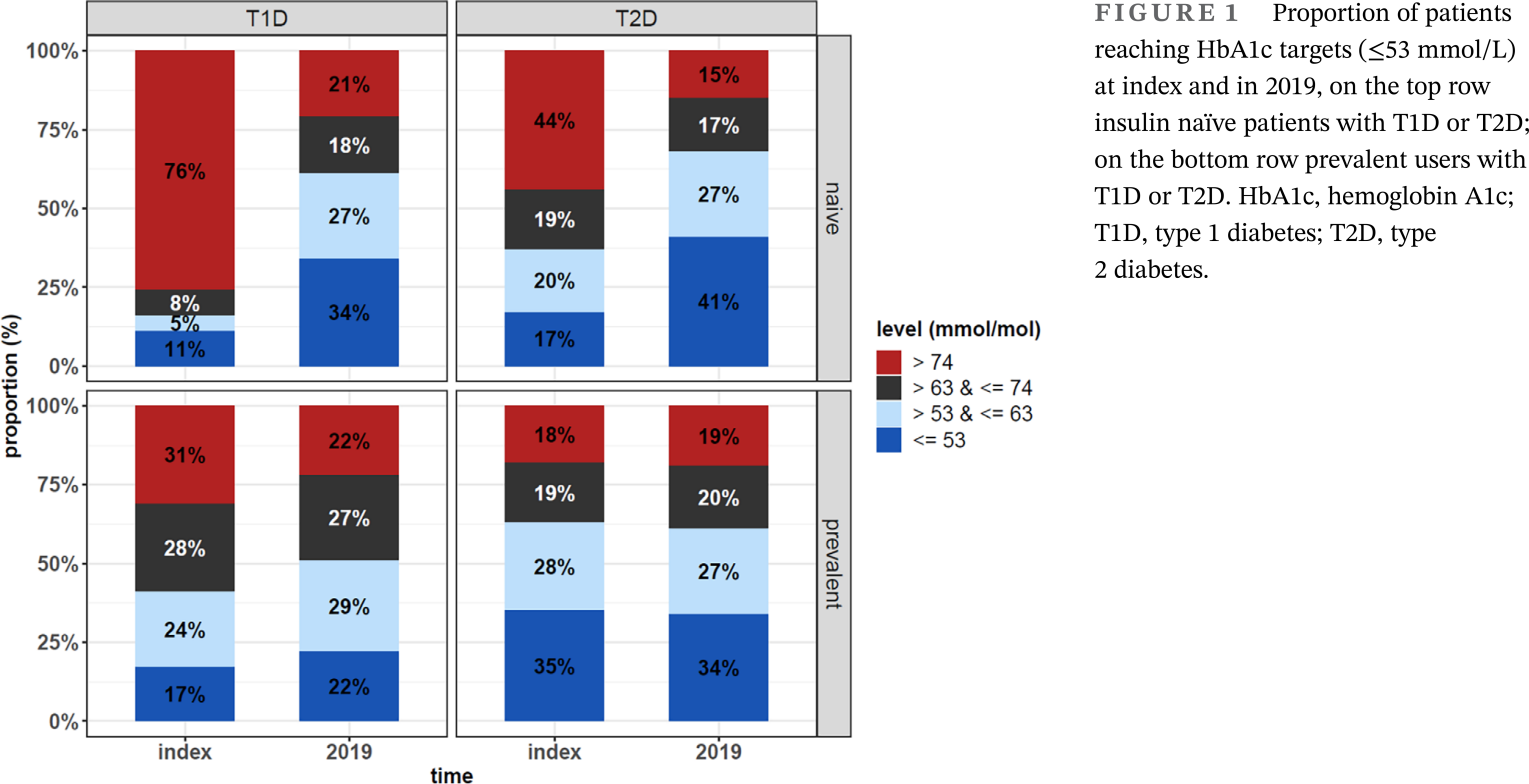

## Graphical abstract

Highlights

- This Finnish nationwide real‐world evidence study used national identity numbers to link data from several registries to identify 145 020 people with diabetes treated with insulin.
- Medication use has evolved with newer generation insulin and noninsulin diabetes medications between 2012 and 2019.
- Despite the aging population, glycemic control of insulin users has remained stable or improved between 2012 and 2019 in Finland.

## 1 INTRODUCTION

Diabetes is a growing global epidemic affecting over 10% of world's adult population.^1^ In Finland, an estimated 429 000 people (total population ≈5.5 million) had diabetes in 2017 (not including people under 30 years) representing 15% of men and 10% of women.^2^ Diabetes is associated with major morbidity and high mortality, and prolonged hyperglycemia can affect several tissues and lead to serious complications if left untreated.^3^ This is especially true within the older demography of patients with type 2 diabetes (T2D), due to their increased comorbidity burden.^4^

Interestingly, Finland surpasses the other Nordic countries (Denmark, Iceland, Norway, and Sweden) in diabetes medication use, especially in the 45–64 age group: 87.3 per 1000 people nationwide in 2019 used diabetes medications in Finland, compared to 54.5–64.2 per 1000 in the other Nordic countries in this age group.^5^ In 2012–2019, second‐generation insulin analogues (insulin degludec and glargine U300 [Gla‐300]) and many noninsulin diabetes medications (such as dipeptidyl peptidase‐4 [DPP‐4] inhibitors, sodium‐glucose linked transporter‐2 [SGLT2] inhibitors, and glucagon‐like peptide‐1 [GLP‐1] analogues) became available.^6^ Although metformin remains the agent of first choice for treating hyperglycemia in most people with T2D, and GLP‐1 analogues are now recommended before initiating insulin, insulin still remains essential for all patients with type 1 diabetes (T1D), severe hyperglycemia, or with T2D when other treatments prove insufficient in managing blood glucose levels.^7^ Nevertheless, the persisting universal problem with insulin initiation in T2D is clinical inertia – introduction of insulin therapy being delayed due to patient‐ or physician‐related factors.^3^, ^8^, ^9^

The Finnish Development Program for the Prevention and Care of Diabetes (DEHKO) project evaluated treatment practices, glycemic control, and population characteristics of a representative cohort of Finnish diabetes population cross‐sectionally at 2000–2001 and 2009–2010.^10^ However, there remains a need for recent detailed clinical data on insulin‐treated patients with T1D and T2D in Finland, especially in the light of the high frequency and fast adaptation of new treatment options. Thus, the primary objective of this study was the characterization of insulin‐treated patients with T1D or T2D in Finland between 2012 and 2019. In addition, insulin treatment patterns and switches between basal insulins (BIs), as well as glycemic control were evaluated. Also, the differences in results between the set index and end of study (EOS), were described.

## 2 MATERIALS AND METHODS

### 2.1 Data sources and data coverage

This descriptive study used existing medical data from various data sources in Finland to investigate the characteristics and comorbidities of individuals with diabetes. People with diabetes were identified based on their insulin purchases and reimbursement rights from the Finnish Social Insurance Institution (SII) Register. Other data sources included the Care Register for Health Care (HILMO), Register of Primary Health Care Visits (AvoHILMO), local primary and specialty care laboratories, and Statistics Finland Causes of Death Register. All data on medication purchases, health care visits and diagnoses, date and cause of death, and selected laboratory results were linked through Finnish personal identity numbers. We attained good geographical coverage of laboratory data but were limited to selected municipalities and regional laboratory providers due to data access limitations. We received laboratory data from the following primary care providers: Turku, Joensuu, Mikkeli, Espoo, and Keusote (includes four cities) that cover ≈14% of total Finnish population. Regional specialty care laboratory providers were HUSLAB (Helsinki and Uusimaa region), TYKSLAB (Turku region), and ISLAB (Kuopio region) that cover ≈40% of total Finnish population. Both primary and specialty care laboratory data were included in the analyses. For example, at least a single hemoglobin A1c (HbA1c) value was available for 27.8% of the entire 145 020 insulin‐treated diabetes cohort and for 15.8% of the entire cohort within 12 months before the index date. The exact numbers of available laboratory test data per study group at index and EOS are shown in Table 1.

**TABLE 1:** Demographic and clinical characteristics, and diabetes‐related laboratory values for patients based on data 1 year before index and EOS (year 2019).

	Naïve users				Prevalent users			
	T1D index	T1D 2019	T2D index	T2D 2019	T1D index	T1D 2019	T2D index	T2D 2019
*N*	4016	3839	47 154	38 031	30 343	27 227	63 507	37 990
Age, years	40.8 (16.2)	44.2 (15.9)	66.6 (13.6)	68.4 (13.0)	45.0 (15.8)	51.4 (15.1)	68.5 (11.8)	72.0 (10.8)
Duration of diabetes in years^a^	N/A	5.0 (3.6)	6.6 (5.6)	10.4 (5.5)	21.5 (13.2)	28.8 (12.8)	11.9 (7.4)	18.7 (6.8)
Male (*N*, %)	2430 (60.5)	2309 (60.1)	27 300 (57.9)	22 290 (58.6)	17 544 (57.8)	15 558 (57.1)	35 918 (56.6)	22 021 (58.0)
Follow‐up time, years	‐	4.1 (2.3)	‐	3.8 (2.2)	‐	7.6 (1.4)	‐	6.4 (2.4)
*Diabetes‐related laboratory values* ^b^								
B‐HbA1c, mmol/mol	102 [76, 125], *N* 1056	60 [49, 71], *N* 773	70 [57, 91], *N* 7536	56 [49, 67], *N* 5119	67 [57, 78], *N* 5905	63 [55, 73], *N* 5461	59 [50, 69], *N* 8406	59 [50, 70], *N* 5581
fP‐LDL, mmol/L	2.8 [2.2, 3.5], *N* 666	2.6 [2.1, 3.3], *N* 620	2.4 [1.8, 3.2], *N* 5330	2.2 [1.7, 2.8], *N* 3728	2.4 [1.9, 2.9], *N* 4716	2.3 [1.9, 2.9], *N* 4495	2.2 [1.7, 2.7], *N* 6727	1.9 [1.5, 2.2], *N* 4256
fP‐trigly, mmol/L	1.3 [0.9, 2.1], *N* 688	1.0 [0.7, 1.4], *N* 589	1.9 [1.3, 2.7], *N* 5187	1.6 [1.2, 2.4], *N* 3170	1.0 [0.7, 1.4], *N* 4648	1.0 [0.7, 1.4], *N* 4157	1.6 [1.1, 2.2], *N* 6643	1.5 [1.1, 2.2], *N* 3597
fP‐total‐C, mmol/L	4.6 [3.8, 5.4], *N* 671	4.5 [3.9, 5.1], *N* 584	4.3 [3.6, 5.3], *N* 5083	3.9 [3.3, 4.7], *N* 3244	4.5 [3.9, 5.1], *N* 4518	4.2 [3.6, 4.9], *N* 4075	4.0 [3.4, 4.8], *N* 6488	3.6 [3.1, 4.3], *N* 3605
Albumin to creatinine ratio, mg/mmol	0.8 [0.4, 1.6], 350	0.5 [0.4, 1.1], *N* 555	1.4 [0.5, 5.1], *N* 2552	1.4 [0.7, 5.9], *N* 2475	0.3 [0.3, 1.1], *N* 1608	0.82 [0.4, 2.5], *N* 3840	0.76 [0.3, 3.4], *N* 1570	2.3 [0.9, 13.9], *N* 2806
eGFR	121 [107, 132], *N* 1321	110 [97, 121], *N* 1210	88 [67, 102], *N* 10181	81 [55, 97], *N* 11182	105 [92, 118], *N* 4525	96 [79, 109], *N* 8926	85 [67, 98], *N* 8863	75 [51, 91], *N* 12165

*Note*: Data not shown for patients classified as other than T1D or T2D. Values presented as mean (SD) or median [25th, 75th quartile]. Abbreviations: eGFR, estimated glomerular filtration rate; EOS, end of study; fP‐LDL, fasting plasma low‐density lipoprotein; fP‐trigly, fasting plasma triglycerides; HbA1c, hemoglobin A1c; T1D, type 1 diabetes; T2D, type 2 diabetes.

^a^ Naïve users: Delay from the initial diagnosis (first date of reimbursement right or diagnosis) to first (index) or last insulin purchase (2019); prevalent users: delay from initial diagnosis (first date of reimbursement right or diagnosis) to January 1, 2012 (index) or last insulin purchase (2019).

^b^ *n* represents number of patients for whom laboratory values were available per test. Closest value within the year before index or EOS reported.

The study was based on the secondary use of health care data and approved by Findata, the Finnish Social and Health Data Permit Authority (study approval number THL/684/14.02.00/2021). No separate ethics statement was required for the study.

### 2.2 Cohort formation

The study cohort included all adult patients with a reimbursement number for insulin (103) and at least two insulin purchases (Anatomical Therapeutic Chemical [ATC] code: A10A*) within 12 months of the first purchase in the years 2005–2019. Maximum follow‐up time was from 1.1.2012 until 31.12.2019 or death, with no minimum follow‐up time used. Data from January 1, 2005 until December 31, 2011 were used to assess baseline information on patients, unless otherwise stated. The start date of January 1, 2012 was chosen considering that the last nationwide characterization of people with diabetes had been performed in Finland in 2010.^10^ Patients were divided into T1D and T2D based on visit‐associated diagnoses given at specialty care (*International Classification of Diseases* code E10 or E11, respectively), and if unavailable, based on diagnoses from primary care. However, many patients (approximately 25%) had records of both E10 (T1D) and E11 (T2D) diagnoses associated with their health care visits. Hence, we implemented cutoff rules of relative diagnosis consistency to determine whether a patient was considered T1D or T2D. These cutoffs were set at 75% for specialty care diagnoses (shown as a histogram in Figure S1C) and 80% for primary care diagnoses. In the end, only 1704 patients or 1.2% of the final cohort were excluded from analyses due to an unclear diagnosis. T1D and T2D insulin users were further divided into naïve users who started insulin after 1.1.2012 and for whom the index was set according to their first insulin purchase, and into prevalent users who had already been using insulin for any number of years before January 1, 2012 and for whom the index was set as January 1, 2012. The cohort formation process has been visualized in Figure S1. The data were analyzed both at the index and EOS.

### 2.3 Statistical methods and data analysis

All the conducted statistical analyses were descriptive in nature. *p*values <.05 were considered statistically significant. No correction for multiple testing was applied. Analyses were performed with R (version 4.0.3; R‐packages tidyverse 1.3.1, survival 3.2–13, survminer 0.4.9, ggalluvial 0.12.3).

#### 2.3.1 Clinical characteristics, medication purchases, and laboratory values at index and EOS

The number of patients and the characteristics were reported using summary statistics (mean and SD; median, first and third quartiles; N and %; where appropriate). Duration of diabetes was calculated based on the first date of the reimbursement number for diabetes (could be before 2005), diagnosis for diabetes, or insulin purchases. The medication usage was assessed from the reimbursed medication purchases up to 1 year before index until EOS. Furthermore, the diabetes‐related laboratory values, including HbA1c, were assessed from laboratory data within 365 days of the index and/or EOS with the closest value reported. Comorbidities were assessed based on all available HILMO and avoHILMO data from 2005 onwards.

#### 2.3.2 Insulin treatment patterns

Treatment persistence was assessed by defining continuous treatment continuum for each patient based on insulin purchases and estimated using Kaplan–Meier analyses. For the analyses, treatment persistence was defined as the duration between treatment initiation and an event, with an event defined as BI switch (measured from the first observed switch), no BI purchase within 7 months after the previous purchase (treatment gap, see definition below), no BI purchase for the rest of the study (treatment discontinuation), or death. EOS was a censoring event. The sequence of different BI treatments and their patterns were assessed and illustrated using Sankey plots. A new treatment was defined as a purchase of a new insulin type in the Sankey plots.

In Finland, a patient can get reimbursed for an amount of insulin that is sufficient for 3‐month usage at a time. To define the treatment gap, median times between the BI purchases (3.5 months; interquartile range [IQR, 2.9, 4.3]) were assessed to justify the length of a grace period that the patients were allowed to have between the purchases, so that the treatment continuum was still perceived to be continued. The 7‐month treatment gap time was selected based on the median time between insulin purchases of 3.5 months plus a 3‐month grace period, confirmed with sensitivity analyses of 5‐, 7‐, and 9‐month gap times.

#### 2.3.3 Glycemic control

All patients with BI purchases and at least one available HbA1c laboratory measurement were included in the analysis of HbA1c outcomes (presented in mmol/mol) at different time intervals. HbA1c values were treated with carry forward imputation, that is, considered unchanged until a new value was available before the following timepoint cutoff (eg, value at 7 months before event would be recorded at 6‐month time point but not carried forward to subsequent time points). Population‐level median values were calculated for patients with T2D before and after insulin treatment initiation, treatment gap, and treatment discontinuation and before and after the first observed BI switch for both T1D and T2D. Patients were categorized into four groups based on HbA1c values: ≤53, >53 to ≤63, >63 to ≤74, and >74 mmol/mol (these mmol/mol values translate to HbA1c glycation percentage values of 7.0%, 7.9%, and 8.9%),^11^ in line with a recent Finnish VALTAVA‐diabetes register pilot study,^12^ with ≤53 mmol/mol as the target value based on the national recommendation.^13^ The significance of the changes in the proportion of patients reaching the target HbA1c (≤53 mmol/mol) in index and in 2019 was assessed with the chi‐square test. The unpaired two‐sample Wilcoxon test was used to assess the significance of the change in the HbA1c values up to 6 months after the event (BI initiation, treatment gap, treatment discontinuation, BI switch) of interest.

## 3 RESULTS

### 3.1 Clinical characteristics, laboratory values, and medication purchases at index and EOS

The insulin use of 4016 naïve users and 30 343 prevalent users with T1D, and 47 154 naïve users and 63 507 prevalent users with T2D was assessed in the study, along with clinical characteristics at index and EOS (Table 1 **)**. T2D naïve users started insulin treatment on average 6.6 years after initial diagnosis. Follow‐up times ranged from 3.81 (SD 2.16) in naïve T2D users to 7.61 (SD 1.35) years in prevalent T1D users.

**FIGURE 1:**
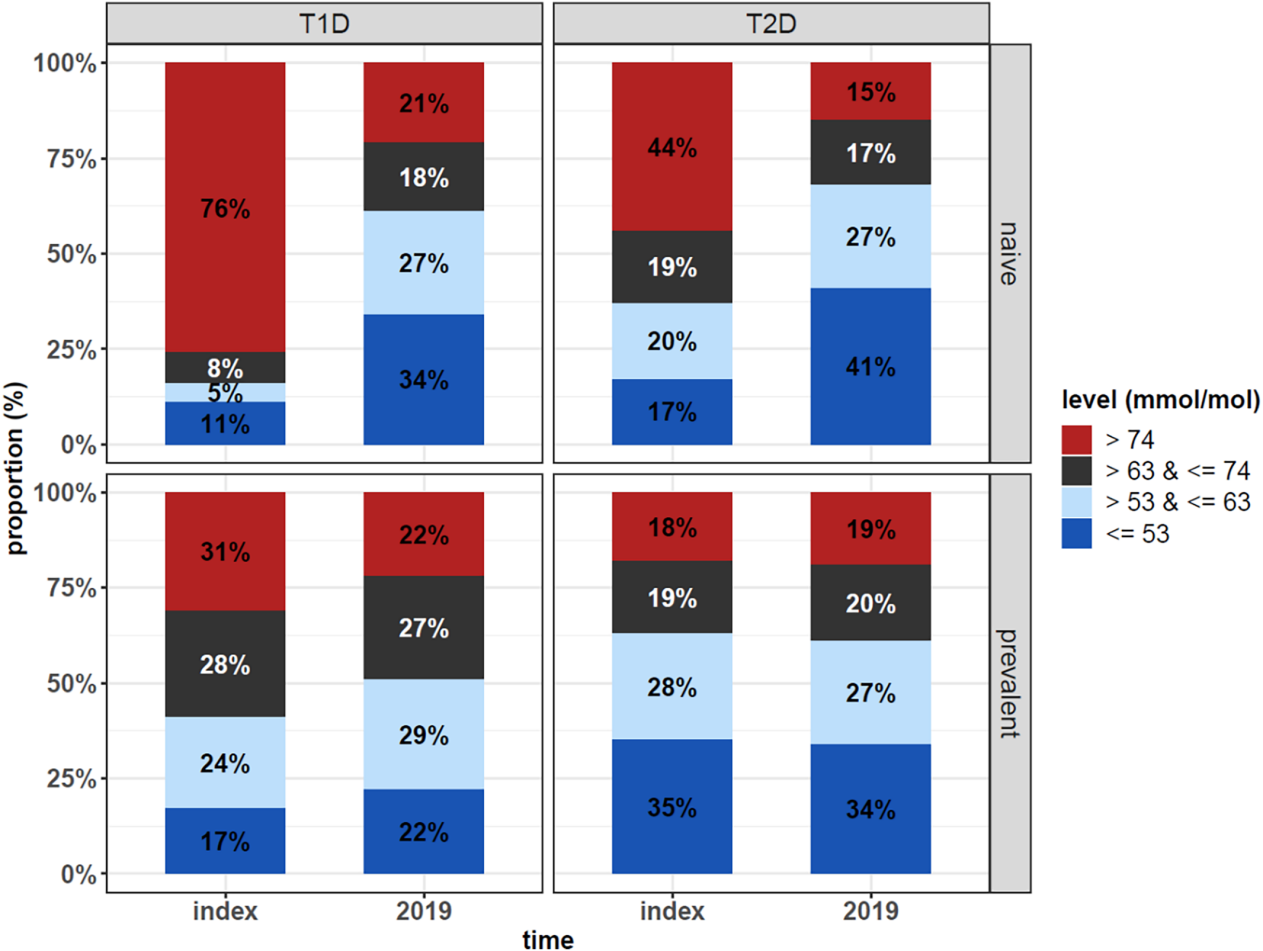
Proportion of patients reaching HbA1c targets (≤53 mmol/L) at index and in 2019, on the top row insulin naïve patients with T1D or T2D; on the bottom row prevalent users with T1D or T2D. HbA1c, hemoglobin A1c; T1D, type 1 diabetes; T2D, type 2 diabetes.

Laboratory values gathered from both primary and specialty care were assessed for a subset of users. The median HbA1c decreased significantly (*p* < .001) from index (70 [IQR 57, 91] mmol/mol) to 2019 (56 [IQR 49, 67] mmol/mol) in T2D naïve users, also reflected in the proportion of users reaching a HbA1c ≤ 53 mmol/mol, which increased from 17% to 41% (*p* < .001). In T2D prevalent users, HbA1c (59 [50, 69] vs 59 [50, 70] mmol/mol) or proportion reaching the target (35% at index and 34% in 2019) remained unchanged (*p* = .129), (Figure 1, right panels). In T1D prevalent users HbA1c was a little lower in 2019 (63 [55, 73] mmol/mol) than at index (67 [57, 78] mmol/mol), but significantly more people reached the glycemic target (22% vs 17%; *p* < .001) and fewer people had a HbA1c > 74 mmol/mol (22% vs 31%; *p* < .001) (Figure 1, left panels).

Diabetes medication purchases at index and EOS are presented in Table 2. The first and second most used insulins throughout the study and in all cohorts were Gla‐100 and detemir. By 2019, prevalent T1D insulin users had been the quickest to adapt Gla‐300 (20%) and degludec (25.9%) insulins. Fast‐acting insulin use was more common in T1D than T2D, as expected. The use of intermediate‐acting neutral protamine Hagedorn insulin decreased in prevalent users through follow‐up, from 7.6% to 2.4% in T1D and 17% to 4.1% in T2D.

**TABLE 2:** Insulin and noninsulin diabetes medication purchases for patients based on data 1 year before index and EOS (year 2019).

	Naïve users				Prevalent users			
	T1D index	T1D 2019	T2D index	T2D 2019	T1D index	T1D 2019	T2D index	T2D 2019
*N*	4016	3839	47 154	38 031	30 343	27 227	63 507	37 990
*Insulin purchases*								
Any insulin purchase (A10A)	‐	3719 (96.9)	‐	35 352 (93)	30 151 (99.4)	26 847 (98.6)	62 259 (98.0)	35 531 (93.5)
Any fast‐acting insulin (A10AB)^a^	‐	3439 (89.6)	‐	8453 (22.2)	28 751 (94.8)	26 116 (95.9)	22 386 (35.2)	17 210 (45.3)
Degludec	‐	824 (21.5)	‐	2333 (6.1)	0 (0)	7062 (25.9)	0 (0)	2618 (6.9)
Detemir	‐	1007 (26.2)	‐	8270 (21.7)	11 178 (36.8)	7649 (28.1)	18 713 (29.5)	8470 (22.3)
Gla‐100	‐	1749 (45.6)	‐	21 808 (57.3)	16 287 (53.7)	7951 (29.2)	31 527 (49.6)	19 114 (50.3)
Gla‐300	‐	519 (13.5)	‐	3655 (9.6)	0 (0)	5450 (20.0)	0 (0)	5383 (14.2)
NPH insulin	‐	28 (0.7)	‐	1095 (2.9)	2311 (7.6)	657 (2.4)	10 823 (17.0)	1553 (4.1)
Only fast‐acting insulin (A10AB)^a^	‐	77 (2.0)	‐	25 (0.1)	1007 (3.3)	1313 (4.8)	68 (0.1)	50 (0.1)
*Noninsulin diabetes medication purchases* ^b^								
Any A10B; *n* (%)	880 (21.9)	580 (15.1)	40 326 (85.5)	33 007 (86.8)	1694 (5.6)	2086 (7.7)	44 458 (70.0)	29 495 (77.6)
Metformin	750 (18.7)	423 (11.0)	30 367 (64.4)	20 508 (53.9)	1461 (4.8)	1467 (5.4)	38 026 (59.9)	19 727 (51.9)
Sulfonylureas	68 (1.7)	<5 (0.1)	9477 (20.1)	420 (1.1)	57 (0.2)	6 (0.1)	5739 (9.0)	222 (0.6)
Thiazolidinediones	23 (0.6)	5 (0.1)	2325 (4.9)	448 (1.2)	30 (0.1)	24 (0.1)	1284 (2.0)	358 (0.9)
DPP‐4 inhibitors	290 (7.2)	117 (3.0)	23 083 (49.0)	14 464 (38.0)	224 (0.7)	122 (0.4)	10 377 (16.3)	9961 (26.2)
GLP‐1 analogues	<5 (0.1)	23 (0.6)	1731 (3.7)	5898 (15.5)	0 (0)	110 (0.4)	0 (0)	6425 (16.9)
SGLT2 inhibitors	65 (1.6)	133 (3.5)	5257 (11.1)	10 163 (26.7)	0 (0)	606 (2.2)	0 (0)	8525 (22.4)
Other A10B	160 (4.0)	79 (2.1)	11 147 (23.6)	6290 (16.5)	172 (0.6)	144 (0.5)	6089 (9.6)	4512 (11.9)

*Note*: Data not shown for patients classified as other than T1D or T2D. Values presented as *n* (percentage in parenthesis). Abbreviations: ATC, Anatomical Therapeutic Chemical; DPP‐4, dipeptidyl peptidase‐4; Gla, glargine; GLP‐1, glucagon‐like peptide‐1; EOS, end of study; SGLT‐2, sodium‐glucose linked transporter‐2; T1D, type 1 diabetes; T2D, type 2 diabetes.

^a^ Denotes all medications under the A10AB group – note that only medication purchase data 12 months prior to index or EOS date included.

^b^ Noninsulin diabetes medications reported at A10B(X) ATC‐code level.

The use of noninsulin diabetes medications (all medications under the ATC category A10B) increased during the study period in T2D insulin users (Table 2). This was due to strong uptake of SGLT inhibitors and GLP‐1 analogues, which was also seen to a small extent in T1D insulin users. At index, 85.5% of T2D naïve users and 70% of T2D prevalent users also used noninsulin diabetes medication. Naïve insulin users used more noninsulin medications than the prevalent insulin users. Metformin and DPP‐4 inhibitors remained the most used medications throughout the study although the number of users declined over time. By 2019 the use of sulphonylurea and thiazolidinedione had become marginal. In contrast, there was a significant adoption of SGLT2 inhibitors and GLP‐1 analogues (respectively 26.7% and 15.5% of T2D prevalent users).

Renal and cardiovascular comorbidities and concomitant medication use are detailed in Table S1. Modest increases in renal and cardiovascular comorbidities were noted between index and 2019. Of note, cardiovascular comorbidities were pronounced in prevalent insulin users: 9.4% of T1D prevalent users were diagnosed with myocardial infarction, ischemic stroke, or unstable angina pectoris by the EOS. Additionally, 9.5% of T2D prevalent users had been diagnosed with a myocardial infarction in the 7 years before index, of whom only 13.9% were alive at the end. Atrial fibrillation also posed a significant comorbidity in both prevalent patients with T1D and T2D at EOS. Concomitant medication use increased moderately between index and 2019 throughout, except for diuretics and other antihypertensives in T2D prevalent users. Particularly in T1D prevalent users the use of lipid modifying agents (index vs 2019: 34.9% vs 48.8%), beta blocking agents (19.1% vs 24.7%), calcium channel blockers (15.9 vs 22.5%), and renin‐angiotensin system medication (41.2% vs 49.5%) increased.

### 3.2 Insulin treatment patterns

**FIGURE 2:**
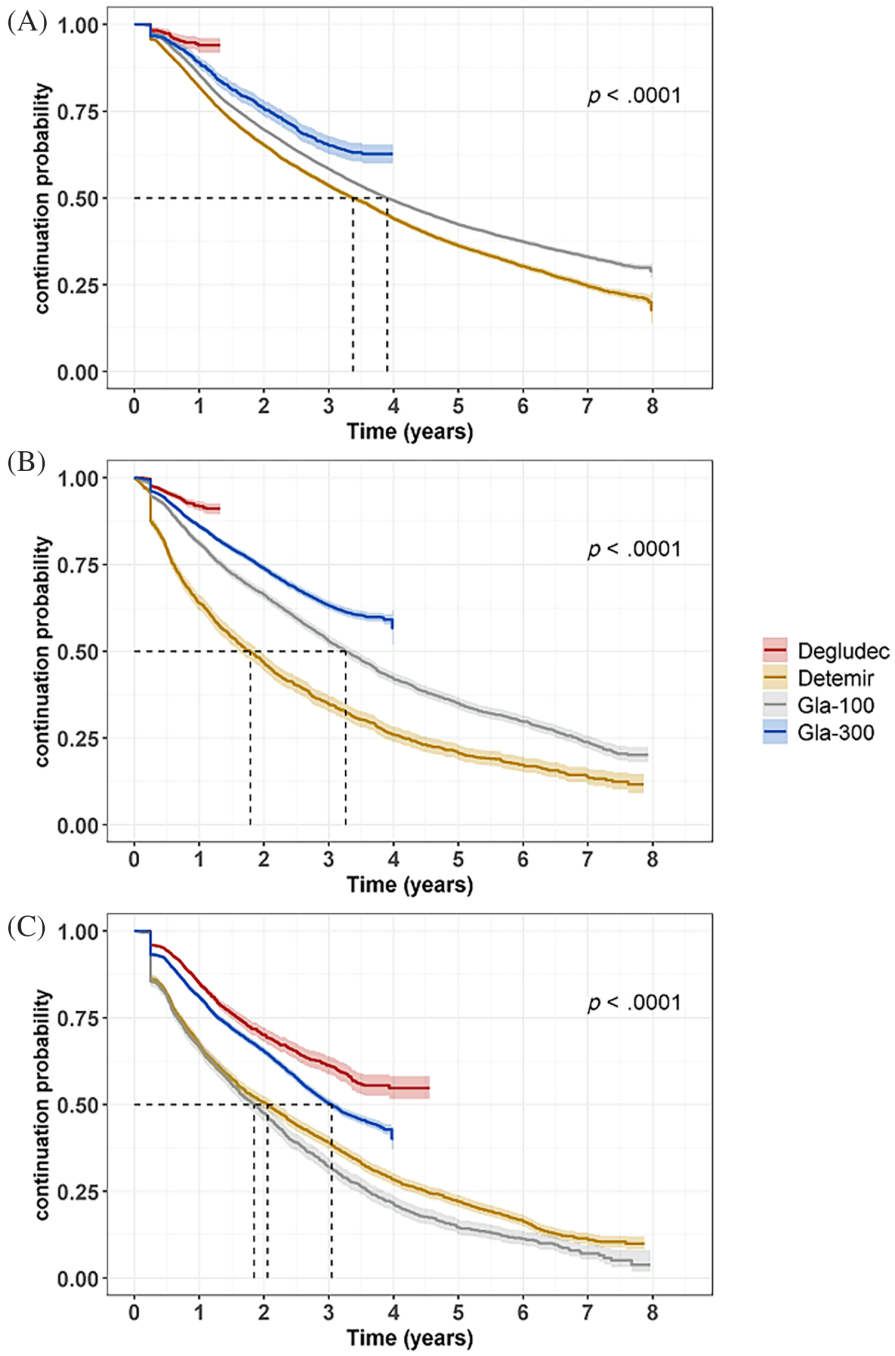
BI treatment persistence for each insulin type in T2D naïve users (A); from first observed switch in T2D prevalent users (B); from first observed switch in T1D prevalent users (C). Insulin purchase gap of 7 months. The follow‐up time for degludec and Gla‐300 was limited, and median persistence thus not reached in all settings. *p* < .0001 denotes significant differences between median persistence with medications reaching a median value obtained using the log‐rank test. Gla, glargine; T1D, type 1 diabetes; T2D, type 2 diabetes.

**FIGURE 3:**
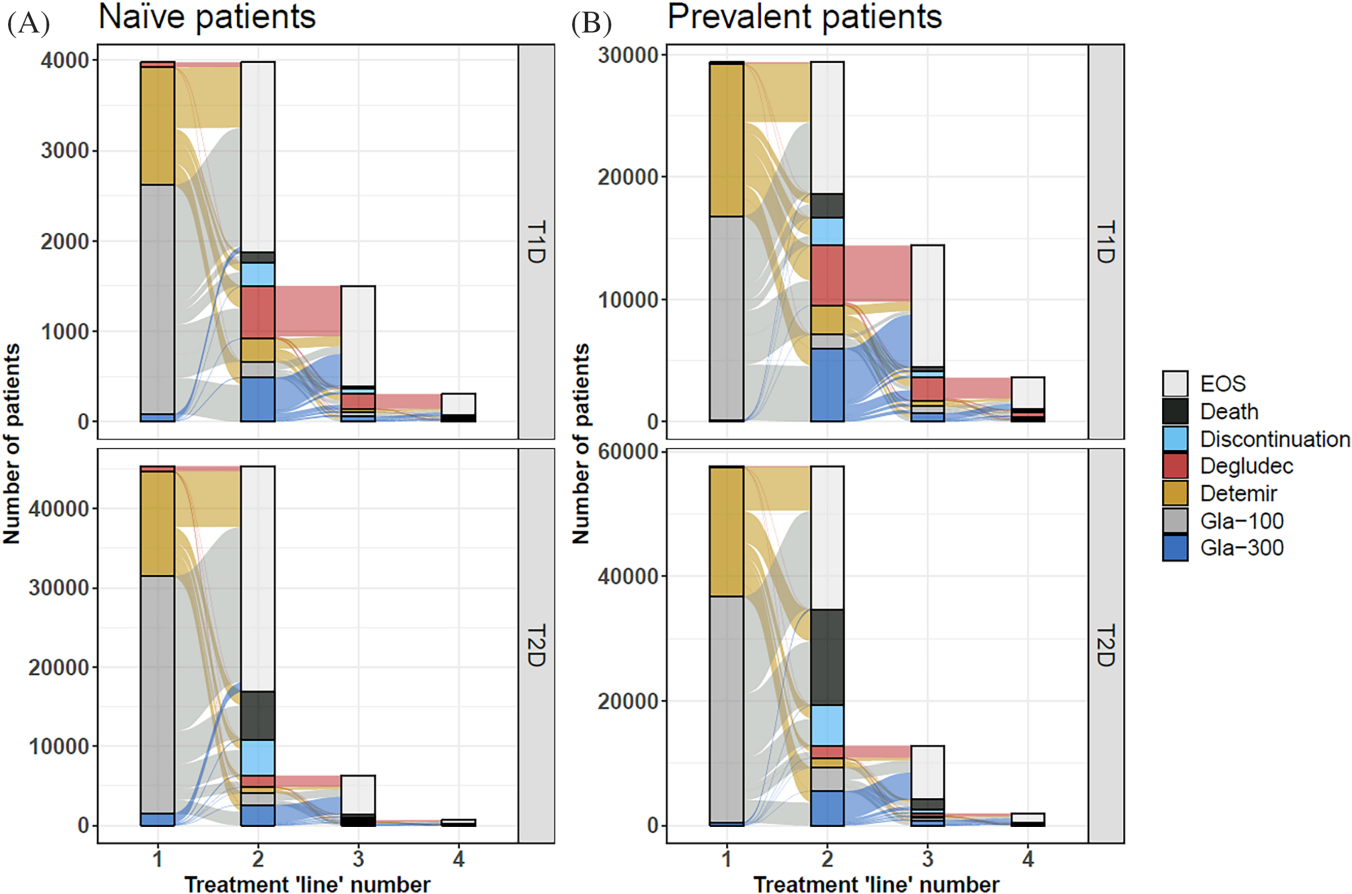
Sankey plot of switches from one BI to another, BI discontinuation, EOS, or death in naïve users (left) and prevalent users (right). Top panels T1D, bottom panels T2D. Please note the scale difference for each panel. BI, basal insulin; EOS, end of study; Gla, glargine; T1D, type 1 diabetes; T2D, type 2 diabetes.

Insulin treatment patterns were described using Kaplan–Meier persistence analyses (Figure 2 **),** Sankey plots (Figure 3), and persistence point estimates (Tables S2 and S3). The median persistence for the most used BI, Gla‐100, was 3.9 years in T2D naïve users and 3.7 years after the first observed switch in T2D prevalent users and 1.8 years in T1D prevalent users. Statistically significant differences were noted between median persistence values using the log‐rank test in all study groups (*p* < .0001), although the follow‐up time for Gla‐300 and degludec was limited, and prevalent users were followed only after the first observed switch. Gla‐100 and detemir were the most common BIs in the first observed treatment line and degludec and Gla‐300 in the second treatment line (Figure 3). In general, people with T2D were less likely to switch from one BI to another and they underwent fewer switches than people with T1D during the follow‐up **(**Figure 3). Observing the persistence point estimates (Tables S2 and S3), at 3 years, T2D naïve users were most likely to stay on Gla‐100 (58%) and least likely to switch (7%) to another BI, and T1D prevalent users were least likely to stay on Gla‐100 (32%) and mostly likely to switch (44%). Treatment gaps of over 7 months were similar in these groups (15%–18%). Discontinuation was low (7% or below), and death occurred in 12% (T2D prevalent users), 10% (T2D naïve users), and 3% (T1D prevalent users).

### 3.3 Glycemic control associated with insulin treatment patterns

**FIGURE 4:**
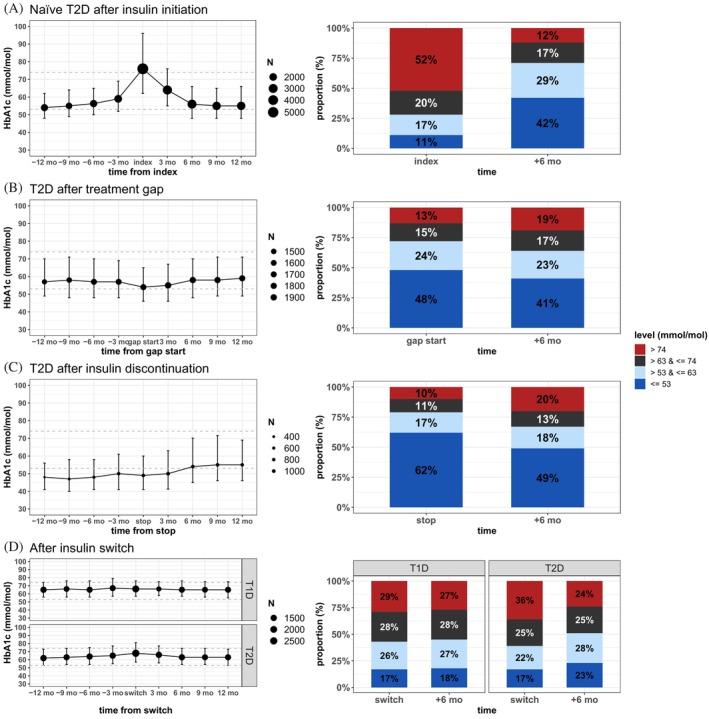
Median HbA1c before and after BI initiation and proportion of patients reaching HbA1c targets in T2D naïve users (A); in people with T2D after treatment gap of over 7 months (B); after treatment discontinuation in people with T2D (C); and after BI switch in people with T1D and T2D (D). For the left panels, the size of the symbol denotes the number of laboratory values available for each data point. BI, basal insulin; HbA1c, hemoglobin A1c; T1D, type 1 diabetes; T2D, type 2 diabetes.

HbA1c levels were assessed in the year before and after the following events: BI initiation in T2D naïve users, treatment gap or BI discontinuation in both T2D naïve and prevalent users, and BI switch in both T1D and T2D (Figure 4 **)**. Total number of unique patients per each event were 45 374 (initiation), 27 425 (treatment gap), 12 012 (discontinuation), 16 960 (switch T1D), and 25 646 (switch T2D). At least one HbA1c value within a year of each event were available for 8536, 5435, 2425, 4393, and 4529 patients, respectively, with HbA1c coverage ranging between 17.6% (T2D switch) to 25.9% (T1D switch) per event.

BI initiation coincided with a peak in HbA1c levels in individuals with T2D. Proportion of individuals achieving HbA1c target (≤ 53 mmol/mol) at 6 months after initiation increased to 42% from 11% at index (*p* < .001; insulin start), whereas individuals with very poor (>74 mmol/mol) glucose control decreased from 52% to 12% (*p* < .001; Figure 4A **)**. Furthermore, there was a significant (*p* < .001) decrease in the median HbA1c at the time of the BI initiation (76 [62, 96] mmol/mol) and up to 6 months after the initiation (56 [48, 66] mmol/mol). Treatment gap had little effect on HbA1c levels in the short term, but fewer patients 41% remained under the HbA1c target after 6 months (*p* < .001; Figure 4B **)**. At insulin discontinuation, 62% were under the HbA1c target, which lowered to 49% at 6 months after (*p* < .001;  Figure 4C **)** and a higher median HbA1c value was reached (49 [41, 63] mmol/mol vs 54 [45, 70] mmol/mol; *p* <.001) at 6 months. No significant changes in HbA1c levels were evident after switches in either group, but the proportion of people reaching the target group in T2D increased to 23% from 17% (*p* < .001; Figure 4D **)**.

## 4 DISCUSSION

In this descriptive nationwide non‐interventional register study, we assessed the clinical and demographic characteristics, medication purchases, BI treatment patterns, and glycemic control in people with insulin‐treated diabetes in Finland between 2012 and 2019. We identified 145 020 people with the reimbursement right for insulin and more than 2 insulin purchases within their first year of insulin treatment initiation using national reimbursement and medication purchase register data. The demographical structure and numbers of people with T1D/T2D confirmed by this study were in line with prevailing literature and statistics, although only insulin users were included in the cohort and the age cutoff was 18 years, which decreased the number of eligible patients with T1D.^12^ Our study showed that glycemic control in insulin users has remained stable or improved between 2012 and 2019, and that both novel insulins and novel noninsulin diabetes medications have been adopted in practice.

The Finnish diabetes population and disease management have been evaluated through several questionnaire‐based studies since the 1990s.^10^, ^14^ A study to evaluate diabetes care in 1993 concluded that glycemic control was poor in the majority of people with diabetes, with a mean HbA1c level of 73 mmol/mol (8.8%) in patients with T2D on insulin treatment alone, and 80 mmol/mol (9.5%) in patients with T2D treated with a combination of insulin and non‐insulin diabetes medications.^14^ In the DEHKO study (2000–2010),^10^ improved disease management results were reported; glycemic control in people with T2D in 2010 with 10–12 years disease duration was comparable to people with T2D with 0–3‐year diabetes duration in 2000, although a rise in HbA1c is normally observed with increased disease duration and age.^15^, ^16^ The median HbA1c further decreased in insulin using T2D population from 67 to 68 mmol/mol (8.3%–8.4%) in 2000–2001 to 61–62 mmol/mol (7.7%–7.8%) in 2009–2010. In our current study, despite the introduction of new medications, the median HbA1c in T2D prevalent users remained stable between 2012 and 2019 (58.5 vs 59 mmol/mol). However, it should be noted that in the same period, average diabetes duration increased from 11.9 to 18.7 years and average age from 68.5 to 72 years. In the T1D population the improvement has been slow, as the median HbA1c in 1993 was 69 mmol/mol (8.5%) and in 2019, 63 mmol/L (prevalent T1D population).^10^, ^14^ Direct comparisons between these studies should be interpreted with caution, however, as our current study focused on the nationwide insulin‐treated adult diabetes population instead of a sample of the entire diabetes population.

Significant changes in diabetes medication use were noted between the study groups and between index and 2019. At a national level in Finland, the SII reimbursement level for insulins (A10A) remained at 100% throughout the follow‐up time in this study, but noninsulin diabetes medication (A10B) reimbursement level changed from 100% to 65% at the beginning of 2017.^17^, ^18^ The introduction of new treatment options, namely GLP‐1 analogues and SGLT‐2 inhibitors (market authorization granted by European Medicines Agency respectively in 2006 and 2013^19^), as well as second‐generation BIs, Gla‐300 and degludec (which entered the market respectively in 2015 and 2013), makes it interesting to compare glycemic control in T2D naïve insulin users at EOS and T2D prevalent users at index. These cohorts have a similar demographic profile (mean age 68.4 vs 68.5 years) and duration of diabetes (mean duration 10.4 vs 11.9 years). Our analyses showed that a larger proportion of T2D naïve users reached the HbA1c target of <53 mmol/mol (41% vs 35%) and fewer remained with poor control (15% vs 18%) in 2019 than T2D prevalent users in 2012. Concomitantly, the use of novel noninsulin medications and BIs was higher in T2D naïve users in 2019 than for T2D prevalent users in 2012. Although this applies no direct causal connection between novel medications and glycemic control, these data seem to support that treatment practices have evolved and adapted into practice between 2012 and 2019, despite a change in the reimbursement level of noninsulin diabetes medication in 2017.^17^, ^20^

Studies assessing real‐world BI use and persistence at a national scale remain rare. In our study, type‐specific BI median persistence was assessed using Kaplan– Meier analyses with respect to treatment gaps, insulin switches, and treatment discontinuations. The median persistence for Gla‐100 and detemir in T2D naïve users was 3.4 and 3.9 years, respectively. Median persistence for Gla‐300 and degludec was not reached within our study period due to limited follow‐up time but was showing better persistence in the first 3 years in prevalent cases and after 1 year in T2D naïve users. Interestingly, this result is in line with a recent study reporting better persistence in people with T2D switching to a second‐generation BI compared to an alternative first‐generation BI.^21^ However, these results must be interpreted with caution because the underlying reasons for the observed treatment patterns and changes in BI use were not explored in this study. Also, persistence was measured only from first observed switch during the study period for prevalent users and may thus be confounded by patients with multiple preceding switches.

Tracking glycemic control through HbA1c levels may provide some clues to reasons behind BI treatment initiation, gaps, discontinuations, or switches, but it is not the sole measure of optimal disease management.^22^ As expected, BI initiation in T2D was effective in lowering HbA1c levels. We observed a slight rise in HbA1c after treatment gaps and discontinuation, with reductions in patients reaching target levels at 6 months, reflecting the role of other interventions, such as lifestyle interventions, intensified noninsulin medications, and gastric bypass surgery. For people with T1D especially, switching to insulin pump and hospital‐administered insulin are likely to contribute to discontinuation figures. We report no significant changes in median HbA1c levels in the 12 months following a BI switch for patients with T1D but a small yet significant change in the proportion of patients reaching the target group in T2D. However, our study did not consider other key factors needed to improve glycemic control, such as titration or adherence. Also, if BI switch occurred due to hypoglycemic events, which remain underreported two‐ to ninefold,^23^ or other adverse effects, less lowering of HbA1c would be expected. These factors are important to note when comparing these real‐world data to randomized controlled trials (RCTs), where HbA1c improvements after switches to second‐generation insulin analogues have been reported.^21^, ^24^

A significant strength of our study was the ability to identify all insulin users in Finland (only excluding the patients treated in hospitals and other institutions) and aggregate and combine their data from numerous nationwide and local registries using unique personal identity codes. By tracking nearly all insulin purchases and diagnoses from primary and specialty care, we could reliably assess demographic data to support accurate T1D/T2D classification and evaluate patterns in insulin use, which remains unique to our study to the best of our knowledge. Not limited by strict inclusion criteria imposed on RCTs, a broader view of overall diabetes management and standard of care at national level is thus gained.

Due to the retrospective nature of our study, however, interpretations of the data should be made with caution. Real‐world data are, by default, partially nonstandardized and have some incomplete parameters. Therefore, some missing values were to be expected and no imputation of the missing data points was undertaken. Although certain associations were observed, causation should not be inferred. A specific limitation was the lack of data on insulin dosage. Also, we were unable to track all hypoglycemias, as many cases tend to be treated outside of health care facilities and thus not reported.^23^ Data on insulin pump use are missing, as the identification of pump users based on the type of insulin purchased is confounded by BI purchased for backup and a small proportion of patients with T2D using only fast‐acting insulin (0.1% in our study). Additionally, due to lack of access and dispersed ownership of data by separate registry holders, that is, primary and specialty care laboratory results, the coverage of patients for laboratory results was somewhat lower than expected (at least on HbA1c value from either source for 27.8% of the entire cohort). However, geographically we have good coverage of south, central, west, and east Finland, and the same current care guidelines^25^ are applied to HbA1c testing throughout. All insulin pump users and most T1D patients are monitored in specialty care, but otherwise patients are mostly monitored in primary care. It is possible that quick HbA1c measurements done at the clinic are not recorded in the laboratory results, causing some lack of coverage. Overall, although not ideal, these data are representative of the cohort.

In conclusion, we successfully provided insight on nationwide insulin use, insulin treatment patterns and glycemic control in Finnish T1D and T2D populations between 2012 and 2019. Glycemic control has remained stable or improved during 2012–2019 despite an aging demographic during a period when many new insulin and noninsulin diabetes medications became available.

## DISCLOSURE

Leo Niskanen has received speaker honoraria from Amgen, Boehringer Ingelheim, Novo Nordisk, Eli Lilly, Sanofi, MSD, and AstraZeneca; research support from Novo Nordisk to the hospital; and has participated in the scientific advisory boards of Amgen, Boehringer Ingelheim, Eli Lilly AstraZeneca, MSD, and Novo Nordisk. Timo T. Valle has received speaker honoraria from AstraZeneza, BMS, Boehringer Ingelheim, SK, Eli Lilly, Takeda, MSD, Mundipharma, Mylan, Novartis, Novo Nordisk, Orion, Sanofi, and Schering Plough and has provided consultation and participated in the scientific advisory boards of AstraZeneca, BMS, Eli Lilly, Janssen/J&J, MSD, Mundipharma, Mylan, Novartis, Novo Nordisk, and Sanofi. Kai Kysenius, Saara Kaijala, and Mariann I. Lassenius are employees of Medaffcon Oy. Minna Hannula is an employee of Sanofi Oy.

## Supporting information


**Supplementary Figure S1.** (A) The cohort formation process. (B) The study group formation process. (C) The distribution of specialty care visit‐associated *International Classification of Diseases, Tenth Revision* (ICD‐10) codes between E10 (type 1 diabetes [T1D]) and E11 (type 2 diabetes [T2D]).
**Supplementary Table S1.** Cardiovascular and renal comorbidities, and concomitant medications of patients at index and end of study (EOS).
**Supplementary Table S2.** Point estimates for glargine 100 (Gla‐100) persistence analyses of naïve type 2 diabetes (T2D), and from first switch in basal insulin (BI) for patients with type 1 diabetes (T1D) and T2D.
**Supplementary Table S3.** Point estimates for insulin detemir, glargine 300 (Gla‐300) and degludec persistence analyses of naïve type 2 diabetes (T2D), and from first switch in BI for patients with type 1 diabetes (T1D) and T2D.
