# Supplementary material for: Trends in clinical characteristics, medication use, and glycemic control in insulin‐treated patients with type 1 and type 2 diabetes in Finland in 2012–2019: Nationwide real‐world evidence study
Source: J Diabetes. 2024 Jan 25;16(5):e13491. doi: 10.1111/1753-0407.13491 (PMC11079632; doi:10.1111/1753-0407.13491)
Supplement: Supplementary file 1 — Supplementary Figure S1. (A) The cohort formation process. (B) The study group formation process. (C) The distribution of specialty care visit‐associated International Classification of Diseases, Tenth Revision (ICD‐10) codes between E10 (type 1 diabetes [T1D]) and E11 (type 2 diabetes [T2D]). Supplementary Table S1. Cardiovascular and renal comorbidities, and concomitant medications of patients at index and end of study (EOS). Supplementary Table S2. Point estimates for glargine 100 (Gla‐100) persistence analyses of naïve type 2 diabetes (T2D), and from first switch in basal insulin (BI) for patients with type 1 diabetes (T1D) and T2D. Supplementary Table S3. Point estimates for insulin detemir, glargine 300 (Gla‐300) and degludec persistence analyses of naïve type 2 diabetes (T2D), and from first switch in BI for patients with type 1 diabetes (T1D) and T2D. [file JDB-16-e13491-s001.docx]

**Supplementary Appendix**

**Supplementary Figure 1**. A) The cohort formation process. B) The study group formation process. C) The distribution of specialty care visit-associated ICD-10 codes between E10 (T1D) and E11 (T2D).

| **A** 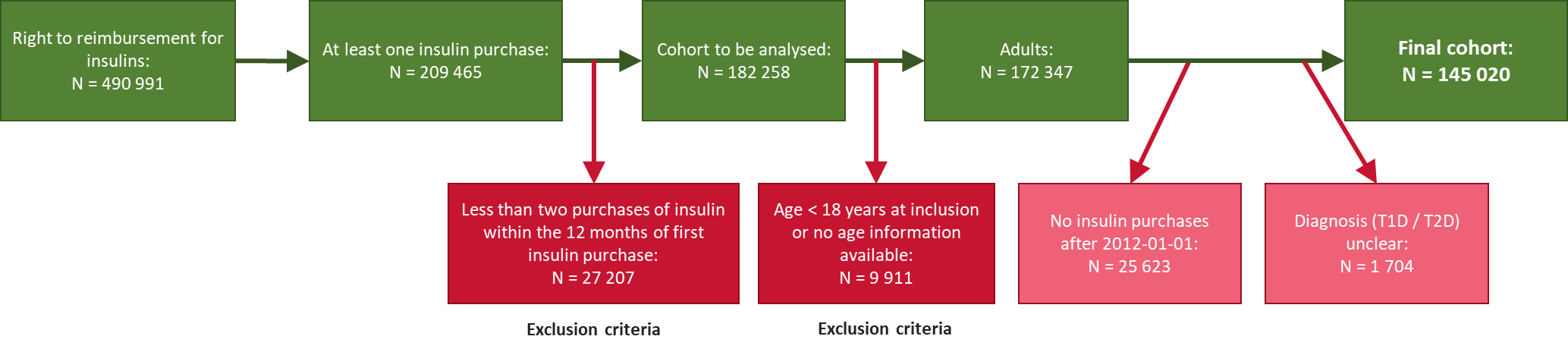 | |
| --- | --- |
| **B** 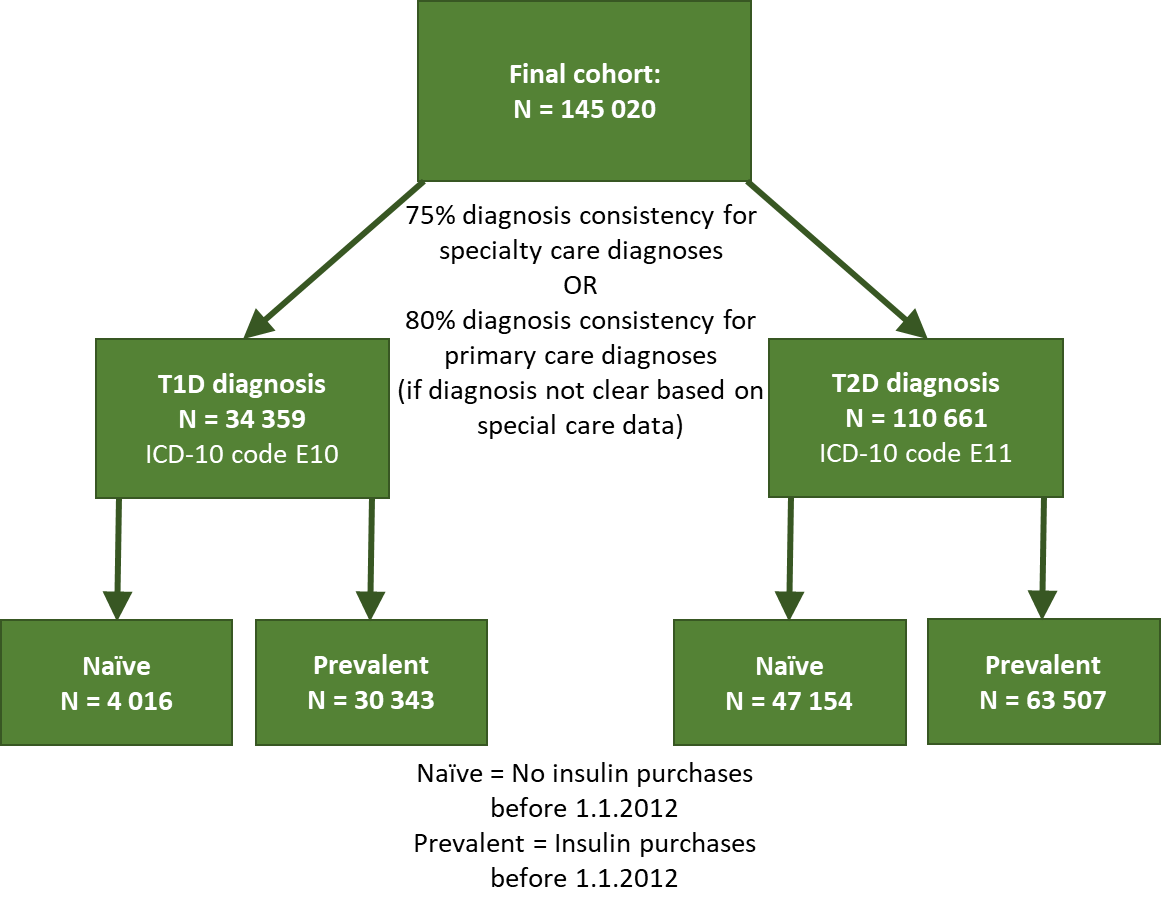 | 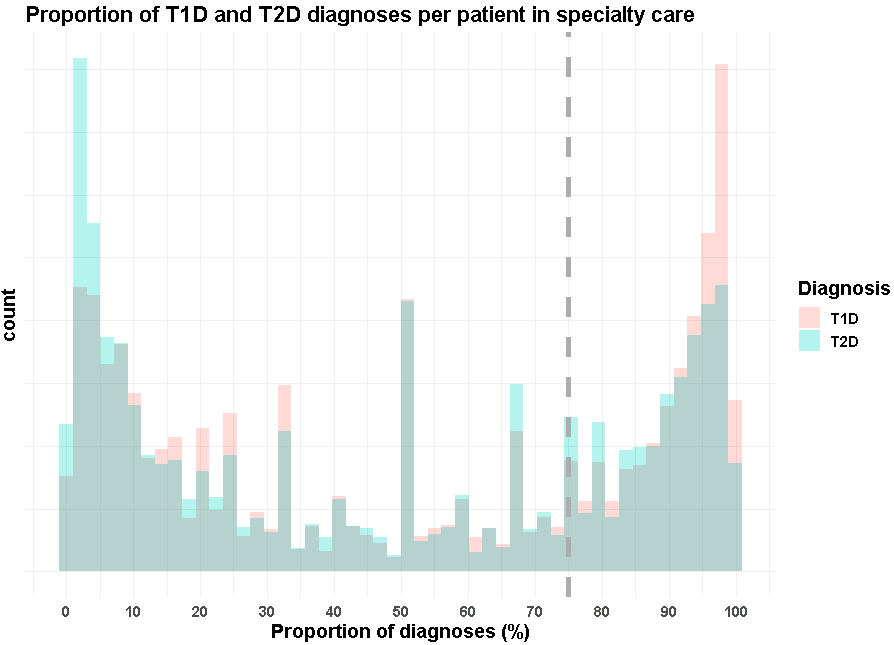  **C** |

**Supplementary table 1**. Cardiovascular and renal comorbidities, and concomitant medications of patients at index and EOS.

|  | Naïve users | | | | Prevalent users | | | |
| --- | --- | --- | --- | --- | --- | --- | --- | --- |
| ***Comorbidity n (%)*** | T1D index | T1D 2019 | T2D index | T2D 2019 | T1D index | T1D 2019 | T2D index | T2D 2019 |
| N | 4016 | 3839 | 47154 | 38031 | 30343 | 27227 | 63507 | 37990 |
| End stage renal disease^1^ | 19 (0.5) | 23 (0.6) | 448 (1.0) | 616 (1.6) | 827 (2.7) | 993 (3.6) | 877 (1.4) | 884 (2.3) |
| Glomerular disorders in diabetes mellitus kidney failure (N08.32)^2^ | 0 (0) | <5 (0) | 17 (0) | 48 (0.1) | 167 (0.6) | 181 (0.7) | 123 (0.2) | 100 (0.3) |
| End stage renal disease or N08.32 (unique patients) | 19 (0.5) | 24 (0.6) | 464 (1.0) | 653 (1.7) | 909 (3.0) | 1056 (3.9) | 967 (1.5) | 957 (2.5) |
| Diabetic kidney disease^3^ | 8 (0.2) | 26 (0.7) | 421 (0.9) | 932 (2.5) | 1634 (5.4) | 1967 (7.2) | 1877 (3.0) | 2036 (5.4) |
| Myocardial infarction^4^ | 35 (0.9) | 54 (1.4) | 3561 (7.6) | 4090 (10.8) | 803 (2.6) | 1493 (5.5) | 6015 (9.5) | 5262 (13.9) |
| Ischemic stroke^5^ | 38 (0.9) | 67 (1.7) | 3196 (6.8) | 3673 (9.7) | 592 (2.0) | 1071 (3.9) | 5171 (8.1) | 4860 (12.8) |
| Unstable angina pectoris^6^ | 13 (0.3) | 18 (0.5) | 1589 (3.4) | 2049 (5.4) | 413 (1.4) | 666 (2.4) | 3498 (5.5) | 2946 (7.8) |
| Myocardial infarction or ischemic stroke or unstable angina pectoris (unique patients) | 75 (1.9) | 126 (3.3) | 7006 (14.9) | 7881 (20.7) | 1495 (4.9) | 2560 (9.4) | 11724 (18.5) | 10131 (26.7) |
| Atrial fibrillation^7^ | 81 (2.0) | 105 (2.7) | 7463 (15.8) | 7085 (18.6) | 526 (1.7) | 1069 (3.9) | 9011 (14.2) | 7937 (20.9) |
| Amputations^8^ | 37 (0.9) | 83 (2.2) | 733 (1.6) | 1190 (3.1) | 585 (1.9) | 1207 (4.4) | 1471 (2.3) | 2143 (5.6) |
| Ketoacidosis^9^ | 705 (17.6) | 770 (20.1) | 580 (1.2) | 680 (1.8) | 2694 (8.9) | 3661 (13.4) | 750 (1.2) | 804 (2.1) |
| Hypoglycemia^10^ | 10 (0.2) | 94 (2.4) | 173 (0.4) | 530 (1.4) | 1139 (3.8) | 2228 (8.2) | 1198 (1.9) | 1698 (4.5) |
| ***Concomitant medication (ATC class)*** |  |  |  |  |  |  |  |  |
| Lipid-modifying agents (C10) | 416 (10.4) | 981 (25.6) | 25360 (53.8) | 24362 (64.1) | 10576 (34.9) | 13281 (48.8) | 42550 (67) | 27240 (71.7) |
| Diuretics (C03) | 102 (2.5) | 122 (3.2) | 12573 (26.7) | 10388 (27.3) | 3003 (9.9) | 2888 (10.6) | 24576 (38.7) | 12846 (33.8) |
| Beta blocking agents (C07) | 384 (9.6) | 441 (11.5) | 24579 (52.1) | 21089 (55.5) | 5783 (19.1) | 6714 (24.7) | 37741 (59.4) | 23086 (60.8) |
| Calcium channel blockers (C08) | 209 (5.2) | 330 (8.6) | 14147 (30.0) | 13551 (35.6) | 4828 (15.9) | 6129 (22.5) | 24013 (37.8) | 16187 (42.6) |
| Agents acting on the renin-angiotensin system (C09) | 524 (13.0) | 925 (24.1) | 29685 (63.0) | 26341 (69.3) | 12487 (41.2) | 13482 (49.5) | 47180 (74.3) | 29128 (76.7) |
| Other antihypertensives (C02) | 8 (0.2) | 15 (0.4) | 1031 (2.2) | 965 (2.5) | 677 (2.2) | 601 (2.2) | 2759 (4.3) | 1473 (3.9) |

^1^dialysis, diagnosis for end-stage renal disease or transplantation; ^2^N08.32; ^3^N08.30 (diabetic microalbuminuria), N08.31 (diabetic macroalbuminuria), N08.39 (unspecific diabetic kidney disease); ^4^I21*, I22*, I25.2, I23*; ^5^I63*, I64*, I65*, I66*; ^6^I20.0; I24.0; I24.8; ^7^I48*; ^8^S78, S88, S98; ^9^E10.1, E11.1, E12.1, E13.1, E14.1; ^10^E16.0, E16.1, E16.2; Procedure codes: NFQ20, NGQ20, NHQ10, NHQ20, NHQ30, NHQ40. Data was assessed from 7 years before index and at EOS.

**Supplementary Table 2**. Point estimates for Gla-100 persistence analyses of naïve T2D, and from first switch in BI for patients with T1D and T2D

|  | T2D naïve users |  |  |  | T1D prevalent users | |  |  | T2D prevalent users | |  |  |
| --- | --- | --- | --- | --- | --- | --- | --- | --- | --- | --- | --- | --- |
| Time (y) | 1 | 3 | 5 | 7 | 1 | 3 | 5 | 7 | 1 | 3 | 5 | 7 |
| **Gla-100** (s0) | 86% | 58% | 42% | 33% | 67% | 32% | 15% | 7% | 81% | 53% | 35% | 24% |
| Treatment switch | 3% | 7% | 11% | 14% | 21% | 44% | 58% | 65% | 5% | 16% | 25% | 30% |
| Treatment gap | 6% | 18% | 24% | 27% | 9% | 18% | 21% | 22% | 6% | 15% | 19% | 22% |
| Discontinuation | 3% | 7% | 9% | 10% | 2% | 3% | 3% | 3% | 2% | 4% | 5% | 6% |
| Death | 3% | 10% | 14% | 16% | 1% | 3% | 3% | 3% | 5% | 12% | 16% | 18% |

**Supplementary Table 3**. Point estimates for insulin detemir, Gla-300 and degludec persistence analyses of naïve T2D, and from first switch in BI for patients with T1D and T2D

|  | T2D naïve users |  |  |  | T1D prevalent users | |  |  | T2D prevalent users | |  |  |
| --- | --- | --- | --- | --- | --- | --- | --- | --- | --- | --- | --- | --- |
| Time (y) | 1 | 3 | 5 | 7 | 1 | 3 | 5 | 7 | 1 | 3 | 5 | 7 |
| **Detemir** (s0) | 82% | 54% | 36% | 25% | 67% | 39% | 22% | 11% | 64% | 35% | 21% | 14% |
| Treatment switch | 7% | 15% | 23% | 29% | 17% | 33% | 45% | 55% | 12% | 25% | 33% | 37% |
| Treatment gap | 6% | 16% | 21% | 23% | 11% | 20% | 23% | 24% | 8% | 14% | 17% | 17% |
| Discontinuation | 2% | 6% | 8% | 9% | 3% | 5% | 6% | 6% | 3% | 5% | 6% | 6% |
| Death | 3% | 9% | 13% | 15% | 2% | 3% | 4% | 4% | 13% | 21% | 24% | 25% |
| **Gla-300** (s0)* | 89% | 65% | - | - | 81% | 50% | - | - | 86% | 63% | - | - |
| Treatment_switch | 3% | 5% | - | - | 7% | 19% | - | - | 3% | 7% | - | - |
| Treatment gap | 5% | 18% | - | - | 10% | 27% | - | - | 6% | 18% | - | - |
| Discontinuation | 1% | 6% | - | - | 1% | 2% | - | - | 1% | 3% | - | - |
| Death | 1% | 6% | - | - | 1% | 2% | - | - | 3% | 10% | - | - |
| **Degludec** (s0)* | 94% | - | - | - | 85% | 61% | - | - | 92% | 83% | - | - |
| Treatment_switch | 3% | - | - | - | 6% | 10% | - | - | 3% | 12% | - | - |
| Treatment gap | 2% | - | - | - | 8% | 24% | - | - | 3% | 3% | - | - |
| Discontinuation | 1% | - | - | - | 1% | 3% | - | - | 1% | 1% | - | - |
| Death | 0% | - | - | - | 0% | 1% | - | - | 2% | 2% | - | - |

**Point estimates not reached within the study period left blank.*
